# Caught on the surface: Tustin on autistic experience

**DOI:** 10.3389/fpsyg.2023.1243310

**Published:** 2023-08-10

**Authors:** Joona Taipale

**Affiliations:** Department of Social Sciences and Philosophy, University of Jyväskylä, Jyväskylä, Finland

**Keywords:** autism, sensory self-absorption, autistic shapes, autistic objects, skin, depth, sense of boundaries, contiguity

## Abstract

According to Frances Tustin, the core of autism is found in sensory modifications—and *tactile* modifications in particular. Tustin argues that sensory experiences may become self-absorbed to such an extent that the sensory environment experientially flattens into a two-dimensional “feel,” which complicates the individual’s relations with the external environment and other people. Focusing on these fundamental modifications and their experiential consequences, the article introduces Tustin’s main insight in terms of collapse of intentional depth, suggesting that this collapse concerns not only *concrete spatial* depth, but *symbolic* and *intersubjective* depth as well. By so doing, the article illustrates how Tustin’s ideas render intelligible certain commonly recognized features of autism, such as “deficits in the ability to initiate and to sustain reciprocal social interaction and social communication” and “restricted, repetitive, and inflexible patterns of behavior, interests or activities” (ICD-11).

## 1. Introduction

The sense of touch plays a central role in our experience: it emerges from the start, matures before the distal senses, and constantly connects us with the external environment. According to Francis Tustin, the lastly mentioned obviousness is challenged in certain cases of autism. Tustin locates the core of autism in modifications of the sense of touch. She argues that tactile experiences can become *self-absorbed* to such an extent that the external environment becomes experientially irrelevant as such: for sure, the environment is all the time “there,” but it appears emphatically or exclusively in terms of the tactile sensations localized on one’s skin. In consequence, the sensory environment is not present as something that exceeds the boundaries of one’s body, but instead comes to equal to this felt “two-dimensional” surface—a modification that fundamentally complicates the connection with the external environment and other people as such.

In this article, I will clarify Tustin’s thoughts on this modification and cash out her core idea in the form of a claim that autism characteristically involves *a collapse of intentional depth*. My exposition is outlined as follows. First, I will examine how touch, in collaboration with the other senses, normally opens into a dimension of depth. When it comes to neurotypical experiences of touch, perceptual attention usually proceeds either *outward* from the surfaces of our touching body, giving rise to an “exteroceptive experience,” or *inward*, making us aware of our physical existence and giving rise to an “interoceptive experience.” Second, I will elaborate on Tustin’s thoughts on the initial, “adhesive identity” of the self, and relate this with the horrifying experiences of *self-dissolution*, *leaking off*, or *falling forever.* Third, I will show how Tustin views autism as a protective reaction to such unthinkable anxieties. Along with the protective maneuver, however, experience collapses into “two-dimensionality”: sensory attention snuggles into the “feel” on one’s bodily surfaces, rather than opening into the exteroceptively given environment or into the interoceptively given bodily self. Finally, I will examine the experiential consequences of this collapse and illustrate the explanatory potential in Tustin’s account. By arguing that the collapse of “depth” covers not only concrete *spatial depth*, but also *symbolic* and *intersubjective depth*, I will suggest that Tustin’s ideas render intelligible certain commonly recognized features of autism, such as “deficits in the ability to initiate and to sustain reciprocal social interaction and social communication” and “restricted, repetitive, and inflexible patterns of behavior, interests or activities” (ICD-11).

Tustin trained as a child psychotherapist in the 1950’s and she published her scholarly contributions between 1968 and 1993. This naturally gives rise to questions on how her theory fits with the more recent findings. While I will not be giving a detailed account on Tustin’s work with respect to the history of autism—from Leo Kanner’s early formulations to the contemporary neuroscientific models and the theory of mind approach (on this, see e.g., Baron-Cohen, 1995; Verhoeff, 2013)—the immense amount of research during the past decades and the transformations in the diagnostic criteria prompt certain preliminary considerations.

For one, while positioning Tustin’s analyses with respect to the present ICD-11/6A02 and DSM-5-TR/F84.0 diagnostics would exceed the confines of the present article, it should be underlined that Tustin’s clinical vignettes primarily refer to cases to be found, in the current diagnostics, at the *severe end of the autistic spectrum*. That is to say, in comparison with the current autistic spectrum, Tustin is thinking of a more narrow clinical category, covering only levels 2–3 of the ASD. While the children she has in mind are characteristically mute or echolalic, do not play, seem withdrawn from social interaction, might show little or no signs of awareness of another-persons as such, display bizarre responses and ritualistic behaviors, and need strong support for basic daily activities (e.g., Tustin, 1992, 3–8, 21–22), Tustin does not really discuss the so-called “high-functioning autism.” Given this focus, it is of utmost importance to keep in mind that her descriptions are not meant to apply *straightforwardly* to all individuals currently diagnozed in the spectrum. On the other hand, Tustin thinks that most individuals manifest autistic features at least in certain respects and momentarily—what counts for her, phenomenologically speaking, is the scope and permanence of these features (e.g., Tustin, 1972, 79; Tustin, 1986, 8, 183, 290). That is to say, in her view, “autistic reactions are an exaggeration of an inbuilt repertoire of elemental reactions” (Tustin, 1988, 44; Tustin, 1990, 90)—something “indigenous in all of us,” but “over-used in […] a massive and exclusive way” in the case of autism (Tustin, 1990, 31; see also Winnicott, 1996, 206–207). Locating autism within a “continuum” (Tustin, 1992, 21)—i.e., considering “autism” as a clinical term describing “the less common extremes of a universal phenomenon” (Winnicott, 1996, 206–207)—puts some pressure on the idea of autism as a “disease” or “illness” (Tustin, 1986, 11; cf. Winnicott, 1996, 206–207), and Tustin is openly worried about clinical approaches that aim at “removing” or “curing” autism (Tustin, 1988, 44; Tustin, 1990, 19). At the same time, the idea of a continuum enables the hypothesis that, *in* focusing on the severe end of the spectrum, one is at once discussing features that figure, to a lesser extent, in the so-called high-functioning autistic persons and neurotypical individuals as well. And so, while Tustin’s characterizations may not be *straightforwardly* applicable to the less severe cases, I hope to illustrate the relevance of her ideas beyond the clinical scope that she herself was primarily working on.

Second, Tustin speaks of “psychogenic autism” (e.g., Tustin, 1986, 17–20; Tustin, 1990, 34–35). While the etiology of autism is today still largely unknown, contemporary research strongly emphasizes the genetic, biological, and neurochemical etiological factors, and the ICD-11 (American Psychiatric Association, 2022) and the DSM-5-TR (World Health Organization, 2022) both classify autism as a neurodevelopmental disorder. As an etiological notion, “psychogenic autism” hence sounds outdated. In particular, it makes it sound as if Tustin was downplaying or ignoring organic etiological factors and *reducing* the etiology of autism to a particular subclass of environmental factors—namely, the psychological environment of the infant. Such a reading would render Tustin’s theory marginal and uninteresting for contemporary readers, as it clearly contradicts with the wide consensus according to which autism is hardly ever caused by environmental factors alone (see, e.g., Rutter et al., 1999; Strathearn, 2009; Sauer et al., 2010; Hermawati et al., 2018). Yet, the term “psychogenic autism” invites an alternative interpretation. For one, while interested in the psychodynamic aspects of autism, Tustin by no means denies the role of the genetic, neurobiological, hormonal, and organic aspects—in her view, the different etiological factors are complementary (see Tustin, 1992, 44). Moreover, Tustin not only repeatedly underlines the complex etiology of autism (Tustin, 1992, 27–28, 32, 45–49) and constantly emphasizes the importance of interdisciplinary cooperation (e.g., Tustin, 1986, 134; Tustin, 1992, 44–49), but she also explicitly claims that “no form of childhood autism can be attributed to purely psychogenic causes” (Tustin, 1992, 45). In the light of such considerations, the concept of “psychogenic autism” cannot be interpreted as a reductionist etiological notion, referring to cases of autism where neurophysiology and genetics play no significant etiological role. In effect, Tustin is forced to revise her initial terminology: she now openly considers the term “psychogenic autism” misleading and ends up replacing it with “psychobiological autism” (e.g., Tustin, 1986, 10–11). As I see it, this brings Tustin’s work out of the margins and enables an interpretation that seems loyal to the direction she was increasingly heading toward the end of her career. According to this interpretation, “psychogenic autism” is not viewed as an *etiological term*, referring to a minor group of cases aside to the major group of “organogenic autism,” but instead as a *methodological term*, denoting a psychodynamic approach to autistic experience—*no matter what the etiology*. I find this interpretation promising, for it blows the unnecessary dust off Tustin’s theory and opens the relevance of her work toward contemporary autism research. And indeed, no matter what the etiological ground, understanding the psychodynamic and phenomenological nature of *autistic experience* is an important task *per se*. In this regard, as I hope to show, Tustin has a lot to offer.

Lastly, due to certain historical burdens, a psychodynamic approach to autism is prone to give rise to alarming questions as to whether Tustin continues the infamous tradition of blaming parents for their children’s plight. Such a tradition was set on already by Leo Kanner, whose initial formulations underlined the “lack of maternal warmth” as a central etiological factor in childhood autism (e.g., Kanner, 1943; Kanner, 1949). However, Tustin is overtly critical toward this hypothesis, which along with Bettelheim’s popularization came to be called as the “refrigerator mother” hypothesis (e.g., Tustin, 1990, 7; Tustin, 1992, 15, 41; see Bettelheim, 1967). For one, notwithstanding her psychodynamic focus, Tustin compares early interactive patterns with a “web of inevitable reactions” to which the parent and the child are both unwilfully caught (Tustin, 1990, 27), and repeatedly emphasizes that early developmental circumstances are so complex, subtle, and unmanageable (Tustin, 1992, 18, 33) that pointing the finger of blame to any particular spot is doomed to be misleading (Tustin, 1992, 15). Moreover, characterizing Kanner’s and Bettelheim’s ideas as “cruel and erroneous” (Tustin, 1986, 65) and rendering them “slapdash diagnoses” that ought to be deplored (Tustin, 1992, 45), Tustin makes it very clear that, in her experience, “[m]ost autistic children have not experienced coldness, neglect or physical violence from their parents” (Tustin, 1992, 33). In short, according to Tustin, autism is “nobody’s fault” (Tustin, 1990, 102, 27; cf. Winnicott, 1996, 212). Tustin’s explicit resignation from the parent-blaming tradition is further underlined by her increasing emphasis on the complex and multifactorial etiology of autism. And our interpretation, where “psychogenic autism” is viewed as a methodological notion, continues along these lines.

All in all, I will focus on Tustin’s ideas with the aim of showing that her conceptualizations open intriguing phenomenological and psychodynamic perspectives on autistic experience. To clarify this, we must first turn to tactile sensibility, the modification of which lies at the core of Tustin’s theory of autism.

## 2. Being in touch with depth

The sense of touch has a central role among the senses owing to its constant, fundamental, and immediate character. These features deserve to be examined one by one. First, touch *continuously* maintains our relation with our surroundings. Be it clothing, solid objects, ground on which we stand, the cold breeze, or the air that we breath, our tactile connection with the environment is something constant. Second, partly owing to this continuity, touch has a *fundamental* status among the senses. To illustrate, whenever we see an object hitting our body but do not feel it by touch, the object appears as a hallucination, something unreal; by contrast, if we tactually feel something that we cannot see, our doubts turn to visual perception—instead of considering the tactile experience as a hallucination, we either question the perceptual circumstances or perhaps consider the object as transparent or invisible. In short, whenever there is a conflict between the two, vision yields to touch. Third, what we touch is, by rule, in *immediate* contact with our sensory surfaces. The contrast with vision and hearing is rather clear. Whereas the latter introduce the object as being at a certain distance, touch presents the object as being in immediate proximity. Unlike with the eye and the seen object, or with the membrane and the heard sound, there is no sensory medium separating the perceiver and the perceived; instead, between the touching and the touched there is an uninterrupted material continuum. In this respect, touch serves as a kind of measure for physical separation: *the absense of touch implies separateness, and the absence of separateness implies touch*. The importance of this insight will be examined later.

However, if the immediacy of touch is thus underlined, do we have to conclude that, literally speaking, we are merely touching two-dimensional surfaces instead three-dimensional things with an inside and depth? The way in which the sense of surface opens to a full-fledged perceptual experience is of course a complicated matter, the full appreciation of which would grossly exceed the confines of this article, but outlining certain facets of it serves our purposes.

For one, to be sure, consecutive tactile impressions are not isolated from each other, but reach beyond themselves and thus form a temporally unitary “feel.” To illustrate this, consider placing your hand on a rock. While moving your hand, at each moment a new bundle of tactile impressions takes over the temporal now-position. However, as the preceding impressions sink into the past, they do not vanish without a trace; on the contrary, each new impression emerges against the background of the preceding impression, which is “retained” in experience, thus constituting an uninterrupted temporal continuum from past impressions to present ones. Likewise, there is a futural horizon to your tactile experience, which is to say that forthcoming impressions are tacitly (though more or less vaguely) anticipated—after all, you might be surprised about the specific quality of an emerging tactile experience, but you are not surprised about the fact that your experience constantly introduces you with new tactile contents. Because of this temporal synthesis, instead of a chaotic sequence of unrelated impressions, what you experience is a continuous surface “feel.” But how does one get from the two-dimensional surface to three-dimensional object, with an inside and depth?

For sure, none of the senses by themselves reaches into the interiors of the rock—the interiors are not sensed by touch, sight, taste, etc.—and yet we experience solid three-dimensional objects rather than just curved two-dimensional surface-formations. Inference and reasoning can be ruled out as well. For we may indeed make rational assumptions concerning *what* lies underneath the surface, but our feeling *that* there is, in the first place, something like an inner space, depth, or “thickness” that can contain something is not a matter of an educated guess. Neither is the sense of depth explained by bodily resistance—for if one were to suggest that the sense of depth emerges along with the realization that the pressed two-dimensional surface resists “upright” movement, one would be readily presupposing the third dimension rather than explaining it. Also, we would be equally begging the question if, in explaining the emergence of depth, we referred to the distinction between “surface touch” (e.g., palpating one’s skin) and “volume touch,” exemplified by cases of palpating one’s bones *underneath* one’s skin (see Katz, 1989, 50–53).

Instead, the experiential pathway from the two-dimensional “feel” into the three-dimensional object is owing to *sensory integration* and *intersensory cooperation*. For one, rather than either seeing or touching the surface at one time, both sense modalities are simultaneously operative. Moreover, instead of mere simultaneity, the senses also inform and build on one another, so that the tactile “feel” is at once imbued with *cross-modal retentions* and *protentions*. To illustrate, while exploring the rock by touch, I can also see, next to my hand palpating the surface, parts of the surface that are not currently touched. This visual information motivates me to explore further with my hand, and the *seen* contour of the surface readily gives rise to *tactile* anticipations—an example of what nowadays are called “affordances” (see Gibson, 1979). In turn, what I currently feel by touch gives rise to visual anticipations: the felt surface currently hidden under my hand is expected to be looking like something. Moreover, besides intersensory protentions, there are intersensory retentions. When exploring the rock, the newly emerging impressions unfold against the background of the impression from another sense modality; tactile impressions come to fulfill visually induced anticipations, and *vice versa*. And so, when *feeling* a knock on my shoulder, I turn to *look* because, by so doing, I expect to gain additional visual information *related to what I just felt*. In seeing my friend’s hand distancing from my shoulder, this visual perception of mine unfolds against the background of my previous tactile perception: I see my friend having just *touched* me.

Such examples illustrate how the tactile “feel” usually emerges within a temporal and multimodal context of perception, and readily both opens toward and emerges against the background of a multisensory object. Differently put, cross-modal protentions and retentions (i.e., intersensory affordances and verifications) render the tactile “feel” as a relative constituent within an intersensory “schema” or a “draft” of the three-dimensional object. In effect, the tactile “feel” does not normally remain isolated from other actual and possible sensations; it *opens a path for them* and *follows their trails*. It is because of such intersensory communication that the tactile “feel” serves as a *pathway* into a multisensory object that is currently perceived in this or that manner, through this or that sense modality, but *affords being perceived otherwis*e—by other senses and by other sensing beings (on this, see Taipale, 2014, 70–86; Taipale, 2019).

While capturing the full complexity of sensory integration, bodily intentionality, kinesthetic self-awareness, and the relationship between action and perception would naturally exceed the confines of this article, the following observation suffices for our purposes. Within a multimodal context, what is perceived from immediate proximity (i.e., by touch) appears as something that *can* also be perceived from afar (i.e., by the distal senses). In this manner, sensory integration contributes to the sense of depth and separation. That is to say, while touch *per se*, in its immediacy, quite concretely *cancels the distance* between the touching and the touched, sensory integration introduces a *sense of separation into the heart of tactile experience*. The touching and the touched are differentiated from one another, and so, rather than *exhausting* the object, the tactile “feel” manifests itself as the “feel” *of* an external object—i.e., an object that may also appear at a distance. In this manner, sensory integration renders the surface “feel” as a *subjective pathway* into the external and hence potentially shared environment.

Moreover, besides an “exteroceptive depth,” tactile experience also makes us aware of the depths of our bodily self. While touched external objects are objects that extend *outward* from our skin, the “interoceptive depth” amounts to a sense of bodily interiority spreading *inward* from our skin, as it were.^1^ The so-called “double sensations” play a role in the realization of our own materiality. After all, we may not only touch objects that are external to our sensing body, but we can also touch the latter and thereby feel the touch in two places at the same time. For example, when squeezing our left hand with our right hand, the place where we feel the compression (i.e., the left hand) also appears as a solid tangible thing that affords squeezing. Along with such experiences, and the “reversible” nature of the situation (e.g., Husserl, 1952, 195), we come to realize that our touching body is also a tangible, concrete spatial entity. This brings us to an important point. Namely, *insofar as such material self-awareness has become part of our experiential reservoir*, the sense of touch can be said to involve a *contiguity of two depths*: namely the exteroceptive depth (i.e., the environment) and the interoceptive depth (i.e., our own body). In this setting, the sensing surface of our body is introduced as a limiting and connecting interface between the *two depths*: our skin both *connects* us with the environment and *separates* us from it.

Here we come to Tustin who argues that autism centrally involves as rupture in the sense of depth. To cash this out, we first need to examine Tustin’s theory of early development.

## 3. Beyond the “two depths”: adhesive identity and unthinkable anxiety

Taking distance to her Kleinian roots, Tustin argues that, during the earliest developmental period, experiences of separation cannot be accounted for within the framework of the “depressive position” and the “schizo-paranoid position” (see Klein, 2018/1946). This is because both readily differentiate between the self and the environment—in our terms, they set out from the adjacency of “two depths.” While in the Kleinian framework, the infant (with interoceptive depth) is allegedly oriented toward exteroceptive depth (the mother’s interiority) from the first, Tustin argues by contrast that the sense of two depths in touch with each other is preceded by a sense of “adhesion” (Bick, 1968; Meltzer, 1975; Tustin, 1990, 15–20) or, as Ogden later puts it, “contiguity” (Ogden, 1989, 155–172). Schematically put, the idea is that before the sense of two depths separated by an interface—before “twoness,” as Tustin puts it (Tustin, 1990, 149; Tustin, 1992, 121)—there is a *sense of interface* without further specifications: “an experience of *a sensory surface* rather than the feeling of *two surfaces coming together* either in mutually differentiating opposition or in merger” (Ogden, 1989, 33; *emphasis added*).^2^ Before a sense of opposition—say, a sense of a “common symmetrical skin between mother and infant” (see Anzieu-Premmereur, 2015, 661)—the “sensory experience *is* the infant” (Ogden, 1989, 35). In a sense, Tustin and Ogden could be said to be here following Freud who famously argues that the primal self is, experientially speaking, a “surface entity,” something that is “ultimately derived from bodily sensations, chiefly from those springing from the surface of the body” (Freud, 1923, 25). In his last unpublished notes, Freud connects back to this issue and writes: “psyche is extended, [but initially] knows nothing about it” (Freud, 1938, 299). Freud’s idea seems to be that, rather than the self always already (*a priori*) finding itself *in* space, which is a Kantian thought, the bodily self and the surrounding space—in our terms, the “two depths”—are initially “projections” of this felt surface (Freud, 1923, 25; Freud, 1938, 299). If these thoughts are combined with Freud’s idea that, subjectively speaking, the self initially “comprises everything” (Freud, 1930, 67), we end up with the view that, according to Freud, *the sense of surface is initially all there is.* That is to say, while still “vertically unintegrated” and hence confined to the present moment (see Taipale, 2023c), the infant’s experience is fully exhausted by the current tactile “feel,” without the latter being as yet arranged as an experience of an individual over and against an object.

Moreover, if the infant’s sense of self is temporarily exhausted by the sense of contact or “equated” with it, an abrupt disruption to the sense of adhesion will quite literally “mark the end of the infant” (Ogden, 1989, 35). According to Tustin, autism can be understood as a “protective reaction” (Tustin, 1992, 18–21, 67) against the “unthinkable anxiety” (Winnicott, 1971, 131) accompanying such self-annihilation. In order to understand Tustin’s notes on the protective function of autism, we should take a look at this specific anxiety that was first conceptualized by Winnicott.

Winnicott argues that the infant’s sense of self is initially “unintegrated” and “multi-nuclear” (Winnicott, 1958, 298; Winnicott, 1988, 116; Winnicott, 1989, 31; Winnicott, 1996, 24; Glover, 2017, 314–320). According to Glover, the infant’s “primitive urges” have a “partial autonomy” (Glover, 2017, 315, 317) and the “body-ego” is thus initially a “loosely organized whole” (Glover, 2017, 277). Winnicott develops Glover’s idea further, and speaks of experiential “bits and pieces” making up the emerging individual (Winnicott, 1996, 24). Arguing that the primal “material” out of which integration sets forth consists in “motor and sensory elements” (Winnicott, 1965, 60; Winnicott, 1996, 24), Winnicott argues that integration in the infant is primarily promoted by *needs* that gather the bits and pieces together (Winnicott, 1988, 117; Winnicott, 1996, 25). And quite concretely so: for example, a nascent feeling of hunger protentionally activates a rich sensorimotor schema, including motor incitements (e.g., sucking reflex), sensory representations (e.g., tactile and visual sensations) and proprioceptive modifications (e.g., sense of being lifted, carried, or held). In Winnicott’s terms, the hungry infant attempts to “create” (e.g., Winnicott, 1971, 15; cf. Taipale, 2021b, 33ff) the feeding situation, and thereby increasingly *functions as a whole*: the “bits and pieces” are thus gathered together, and there is, for a short moment, “a self to be aware” (cf. Winnicott, 1958, 98; Winnicott, 1988, 117; Winnicott, 1996, 25). Importantly, if the need is satisfied without too grand a delay, what is realized is not just the need but also *the nascent sense of self* that was given rise by the need. In this sense, moments of *need-fulfilment* are at once moments of *self-realization* (see Taipale, 2023c). Assuming good-enough care, such moments are countlessly repeated during the first weeks of the infant’s extrauterine life, and this gradually stabilizes their sense of self.

Accepting the infant’s “absolute dependency” on the caregiving environment, and thereby subscribing to Winnicottian externalism, Tustin argues that the child’s sense of being not only *presupposes* an actual caregiving environment but also *substantially includes* contact sensations with the latter. Consider the feeding situation and the respective mouth/breast or mouth/bottle experience from the infant’s point of view: *the tactile composition* of such repeated formative experiences is presumably soon taken for granted, much like we all take for granted the nearly constant contact sensations provided by our tongue when in touch with the mucosa of our mouth and teeth. In its habitual guise, the mucosa does not stand out as something *over against our touching tongue*, but the sense of contact is rather arranged as a taken-for-granted segment in *how our tongue feels like*. And so, if this defining surface would suddenly be absent, our awareness of our tongue would be radically altered—along with the absence of a sensory “container,” the “contents” seem to lack a defining surface. Likewise, the infant’s sense of self, as introduced by the felt hunger, may protentionally include contact sensations with the caregiver. Instead of confusing “mother” with “herself”—which would refer to the Kleinian “schizo-paranoid position”—here we are dealing with *an adhesive sense of being:* what is included as a constituent of the infant’s sense of being is neither the caregiver nor parts thereof, but a constellation of contact sensations with the latter.

Now, if the sense of contiguity with what actually is something external may initially contribute to our sense of boundedness and definition, the absence of the sense of contiguity will entail something much more terrifying than the absence of an external object—namely, *the horror of lacking a defining surface*. Winnicott addresses this issue by noting that the experiences of “gathering together of the bits” are “dangerous moments” or “precarious states” (Winnicott, 1958, 226; Winnicott, 1965, 145; Winnicott, 1988, 117). Namely, if the infant’s need is not met in due time, the initial “sensation ego” (Tustin, 1992, 22)—namely *the nascent sense of self* tentatively introduced by the need—is *disintegrated* or *dissolved* (see Winnicott, 1958, 98; Winnicott, 1988, 60). This is why losing the sense of contiguity may give rise to unthinkably horrid feelings of “leaking off,” “falling to pieces,” or “breaking apart” (e.g., Winnicott, 1989, 128, 139, 187, 572). Tacitly criticizing the Kleinian framework, Winnicott notes that the respective sense of loss is “of a more obscure kind than is the case with reactive depression and derives from an earlier date in the development of the individual” (Winnicott, 1965, 222):

“For example, the loss might be that of certain aspects of the mouth which disappear from the infant’s point of view along with the mother and the breast when there is a separation at a date earlier than that at which the infant had reached a stage of emotional development which would provide the infant with the equipment for dealing with loss. The same loss of the mother a few months later would be a loss of object without this added element of a loss of part of the subject” (Winnicott, 1965, 222).

Tustin’s famous case with “John” resonates with Winnicott’s example:

“4-year-old John saw his mother’s friend feeding her baby at the breast. This made a great impression on him and stimulated him to tell me about what he referred to as ‘the black hole with the nasty prick.’ This was John’s attempt to put into words an experience he had in early infancy, when he had no words to conceptualize it. It was not exactly a metaphor. This picturesque phrase picked up the essential essence of the original experience, for ‘holes’ are something we have an inbuilt reaction to avoid, and ‘pricks’ are something that we flinch from. John conveyed to me that this ‘black hole’ experience was the result of his finding, as a very young baby, that the nipple of the breast, or teat of the bottle, the ‘button’ as he called it, was not part of his tongue or his mouth, but was separate from it and thus was not under his control. He felt that it had broken off and been lost in a traumatic way, turning the mouth into ‘a black hole with a nasty prick”’ (Tustin, 1990, 78–79; Tustin, 1972, 4–31).

That is to say, by realizing the separateness of what initially had been an integral segment of his bodily self—the tactile “feel” owing to mouth/breast contiguity—John felt to be losing part of the defining surface that secured his unity. As a consequence, he felt he was lacking an “important bit of his tongue” (Tustin, 1990, 148–149).

Certain incoherences in Tustin’s conceptualization are worth noting. Namely, in the current context, it seems misleading to talk about “separateness,” about “losing parts of oneself,” and so on. After all, what is felt to be lost according to Tustin (and Winnicott) is not, say, the breast or part of the mouth, but *the tactile feeling of contiguity and boundedness* of the sensation ego. The idea of a solid self-standing over and against a solid object, with segments being transferred from one to the other, fits with the Kleinian framework of thinking, which sets out with the adjacency of “two depths,” but it seems less suitable for Tustin’s purposes. Realizing this, Tustin writes: “the formulations derived from my cloistered Kleinian training, which had stood me in such good stead when working with other patients, did not adequately encompass the phenomena I was encountering when working with autistic children” (Tustin, 1990, 79). While we may assume that Tustin’s occasional conceptual incoherences in this respect are at least partly owing to her Kleinian training, we ought to keep in mind that whenever Tustin speaks of “separateness” in this connection, it is not the loss of *an object or a part-object*, but the loss of *a defining surface*, that it at issue.

The absence of a felt surface that would hold, bind, or contain one’s sense of being, gives rise to horrifying feelings of “leaking off” into “an engulfing ‘nothingness”’ or “spilling out” into an “endless void” (e.g., Tustin, 1986, 127–128; Tustin, 1990, 218). Indeed, unpleasant feelings of losing the sense of body boundaries are not uncommon among autistic individuals (see Grandin, 1995, 25).^3^ Tustin argues that, in the face of such “existential threat” (Tustin, 1986, 25; Tustin, 1990, 39), the individual reacts by producing the needed sense of adhesion by oneself. Like a psychic equivalent of the *moro reflex* perhaps, one reactively tries to gain physical contact with something concrete that would provide a sense of orientation, boundedness, and security. Tustin locates the psychodynamic function of autism in this attempt to maintain and secure the individual’s adhesive self-definition, “an ever-present tangible link” (Tustin, 1972, 27), which protects the self from the “recurrence of the conditions of the unthinkable anxiety” (Winnicott, 1996, 221). The *protective function* of autism will be studied in the following.

## 4. Sensory dead ends: “autistic shapes” and “autistic objects”

Tustin argues that, as a reaction to the threatening “unthinkable anxiety,” autistic individual takes a *refuge to sensory self-absorption*. Sensory self-absorption is also a normal phenomenon that typically emerges in connection with particularly intensive sensations, whether pleasant or unpleasant. To illustrate, consider how you would feel if someone unexpectedly poured a bucket of icy water on you: for a moment, your experiential reality includes nothing but this shocking sensation; you are wholly captivated by the “feel”—you *are* it, as we saw Ogden putting it. In this manner, sensory self-absorption efficiently clouds your intentional awareness of the world—instead of focal awareness of cold water touching your body, or of your body touching the cold water, your focus currently resides in the sensory “feel.” To be sure, as the shock soon passes, your attention quickly turns toward the cause of your sensation, or toward your soaking body, whereby you may contextualize the shocking sensory experience and evaluate it in the light of its cause.^4^ However, while sensory self-absorption is typically a fleeting and hence relative state, in autism it can be *more frequent and global*—and also something one *actively pursues* and *maintains.* In autistic self-absorption, external objects may not only be temporarily *clouded* by the “sensory feel,” but altogether *equated* with it (e.g., Tustin, 1990, 17). And so, while the sensory “feel” typically serves as experiential *pathway* into an exteroceptive depth or into an interoceptive depth, in autistic experience it may become a “dead-end” (Tustin, 1986, 63; Tustin, 1990, 102): a path leading nowhere.

To illustrate how intentional depth is compromized in such cases, we need to take a closer look at what has thus far been called the sensory “feel.” Tustin elaborates this by distinguishing between “autistic sensation shapes” and “autistic sensation objects.” These two notions deserve a closer look.

### 4.1. Autistic shapes: sensory tranquillizers

“Autistic shapes” are “vague formations of sensation” that “offset the randomness of the flux of sensations which constitutes the infant’s sense of being” (Tustin, 1986, 121). The first shapes arise “without the child’s intervention,” but “the child will soon learn that he can make some ‘shapes’ recur by his own movements. Thus, as well as arising spontaneously, they will become self-induced” (Tustin, 1986, 121). In contrast to “objective visual shapes,” they are “tactile endogenous swirls of sensation” that “distract attention away from unbearable bodily separateness” (Tustin, 1992, 19, 42). Given that the sense of “separateness” to be avoided is now understood in terms of “leaking,” the protective function of autistic shapes can be seen in their capacity to produce sensory experiences that promote the sense of security and boundedness. As such, autistic shapes function as “tranquillizers” (e.g., Tustin, 1990, 100); they are “like a self-induced warm bath that is always on tap” (Tustin, 1986, 128) or “a kind of autogenerated hypnosis which makes the child feel safe and comfortable” (Tustin, 1992, 20).

According to Tustin, the earliest shapes “arise from the ‘feel’ of soft *bodily substances* such as feces, urine, snot, spit, the food in their mouths, and even vomit, some of these being elements for repeated experiences” (Tustin, 1986, 121–122): autistic shapes commonly “arise from soft bodily sensations, such as the flow of urine from the body, or bubbles of spit around the mouth” (Tustin, 1990, 99). Tustin coherently emphasizes that what is important here is not the substance itself, but the tactile contour and pattern produced by it: “bodily substances are merely shape-producers” (Tustin, 1986, 121). This also applies to *bodily self-movement* which increasingly expands the child’s shape-producing possibilities. Sensory shapes can also be produced by spinning and rocking, for instance. Again, what is important is not the movement itself, but the sensory “feel” gained by it. Finally, shapes may also be produced by palpating *physical objects, materials, and textures*, though not attended as something external: “autistic ‘shapes’ were also produced by non-bodily objects and processes experienced as if they were bodily ones” (Tustin, 1986, 122). This relates to what is generally known as “stimming”—the common repetitive activity in autistic individuals toward their special objects of interest, which primarily aims at producing certain kinds of sensory feeling.

Indeed, to some extent, most of us unconsciously engage with such idiosyncratic “shape-producing” activities (see Tustin, 1986, 132): some might be used to pinching and pressing their hands while speaking, some chew their nails when nervous, some tend to drum with their feet while reading, some are accustomed to turning their ankle in a particular manner while watching the television, some habitually fumble their lips while lost in thought, some have their familiar rocking chair where they relax, and so on. Here, too, the ensuing sensory niches are generated for purposes of soothing, enhancing focus, providing a sense of orientation, and so on. The difference with autism is that such shape-producing activity is more global, “a perpetually dominant state” (Tustin, 1986, 132). As the autistic author Temple Grandin exemplifies:

“[As a child] I enjoyed whirling around, and spinning coins and lid jars around. Deeply absorbed in the spinning movement, I did not see or hear anything. People around me were transparent. No sound entered my world. It was as if I was deaf. Even a sudden loud noise did not startle me out of my world. [Yet], when back in the world of human beings, I was extremely sensitive to noise” (Grandin, 1986, 25).

“Rocking and spinning were […] ways to shut out the world when I became overloaded with too much noise. Rocking made me feel calm. It was like taking an addictive drug. The more I did it, the more I wanted to do it. My mother and my teachers would stop me so I would get back in touch with the rest of the world” (Grandin, 1995, 34–35).

Though there are degrees to this for sure, here sensory self-absorption is not something one does while doing something else, but more of a goal in itself.

Moreover, Tustin argues that basically anything can serve as a sensory “shape-producer” (Tustin, 1986, 137). This gives the use of autistic shapes a rather global character. Tustin interestingly exemplifies her concept by asking the readers to attend their tactile “feel” when sitting on a padded chair:

“Try a little experiment. Forget your chair. Instead, feel your seat pressing against the seat of the chair. It will make a ‘shape’. If you wriggle, the shape will change. Those ‘shapes’ will be entirely personal to you. The autistic child’s attention becomes so focused upon these entirely personal ‘shapes’ that the chair, as such, is not important to him, although he may be vaguely aware of its existence” (Tustin, 1986, 125).

In normal development, “the integration of tactile and visual perceptions takes place in the first few days of the infant’s life” (Tustin, 1986, 62); “the integration of tactile and visual sense impressions, and thus the awareness of three-dimensionality, is [normally] present almost from birth” (Tustin, 1990, 53). However, the task of “forgetting the chair” proves very difficult. This is because the tactile “feel” at once arranges itself as a partial appearance of an object that also affords a visual representation (Tustin, 1986, 105, 121). Admitting the difficulty of ignoring the chair in its three-dimensional, practical, and cultural significance and attending exclusively on the sensory “feel” given rise by it, Tustin writes:

“We live in a world dominated by words and by the shapes of actual objects. In studying autistic children we have to try to enter a wordless world dominated by self-induced, amorphous, unclassified, concocted ‘shapes.’ Writing this chapter has brought home to me how difficult it is to cross the threshold into this world. The reader may be finding it as difficult as I did” (Tustin, 1986, 125).

Moreover, besides opening toward the multisensory object-representation, the tactile “feel” also normally “consults” the latter, because of which we are inclined to interpret our tactile “feel” in the light of our grasp of the multisensory object. To illustrate, when sitting down and characterizing our tactile “feel,” we tend to use descriptions that are borrowed from our representation of the multisensory, three-dimensional, and hence intersubjectively verifiable features of the padded seat: we may say, for instance, that there is a “pitted” or “curved” character to our tactile “feel.” And so, while the tactile “feel” is “entirely personal,” it normally introduces itself within a multisensory three-dimensional context. And so, while reflecting upon our tactile “feel,” our intentional relation with the external multisensory object is constantly retained.

According to Tustin, this intentional pathway from “sensation shapes” to the potentially shared objects is compromized in autism (Tustin, 1986, 121). Instead of being organized as a partial and relative appearance *of* the multisensory object, the sensation shape now assumes a focal position, which complicates awareness of the multisensory object as such. In the light of our earlier note that sensory integration introduces a sense of separation into the heart of tactile experience—into the heart of the sensory modality that “obliterates” separateness (Tustin, 1990, 171) and promotes “concrete ‘no-distance’ experiences” (Maiello, 2015, 40)—we may recognize, in sensory self-absorption, a *protective function* (see Tustin, 1990, 17–18). After all, as the process sensory integration is disturbed, what is also avoided is the sense of separateness (see Tustin, 1990, 41). In this manner, the complication of sensory integration may be viewed as a “protective reaction” (Tustin, 1990, 18–19, 218; Tustin, 1992, 18). Interestingly, Tustin herself explicitly avoids characterizing autism in terms of a “defense mechanism” (e.g., Tustin, 1990, 154). Despite the structural similarities, what she has in mind is something more elementary, something “proto-mental” (Tustin, 1992, 18), and something “much more devastating than ‘denial”’ (cf. Tustin, 1986, 132; Tustin, 1990, 109): “Autistic encapsulation seems to be an elemental concretized forerunner of ‘repression’, of ‘denial’ and of ‘forgetting’. I see it as being a psycho-physical protective reaction rather than as a psycho-dynamic defence mechanism” (cf. Tustin, 1986, 153, 309; Tustin, 1990, 154, 43). In this connection, Tustin also notes that “such phenomena fall outside the range of current [sc. Kleinian] psychoanalytical formulations” (Tustin, 1990, 95). Because of this conviction, Tustin widens the psychoanalytic framework beyond the setting that we have referred to by talking of the “two depths,” while increasingly stressing the importance of interdisciplinary cooperation (e.g., Tustin, 1992, 45–50).

Tustin argues that, as “inchoate tactile manifestations” (Tustin, 1986, 142), autistic shapes are “not related to the shapes of actual objects” (Tustin, 1990, 18).^5^ Instead of opening awareness outward, these idiosyncratic “whorls of auto-generated sensations” either “stultify” (Tustin, 1992, 171) or altogether “deaden” (Tustin, 1990, 18) awareness of external reality as such. Correspondingly, objective descriptions do not seem to capture the idiosyncratic “feel.” Instead of simply appearing inadequate or inaccurate, such descriptions seem *empty*: they boil down to formal placeholders for idiosyncratic content, much like the word “this” in saying that “I feel like this.” Likewise, saying that there is a “circular” or “pitted” “feel” to the chair would merely awaken the question how “circular” or “pitted” feels like. Hence the articulation of autistic sensation shapes tends to give rise to idiosyncratic “ordering principles” (Tustin, 1986, 128) that seem “meaningless to the ordinary observer” (Tustin, 1990, 17–18). Indeed, others might understand *that* the ensuing an idiosyncratic categorization system is important to the individual, and value it as such, but they are nonetheless “unshared” and hence remain a kind of “private language”:

“In normal development, this shape-making propensity will soon become associated with the actual shapes of actual objects. This will result in the formation of percepts and concepts which facilitate a working relationship with objects in the outside world which can be shared with other people. […] In autistic children, the shape-making propensities have taken an atypical course which seriously hampers ongoing psychological development. Because their ‘shapes’ are unshared with other people, they become entirely personal and peculiar. They are much more contrived than those of normal children” (Tustin, 1986, 121–122).

Tustin compares normal sensation shapes with *sensory working models* or *working simulations* (Tustin, 1986, 100, 111–112, 124), which are increasingly *adjusted to* and *aligned with* the object itself and hence function as “rudiments for emotional, aesthetic and cognitive functioning” (Tustin, 1986, 121). In autism, the “feel” is no longer *bound* by intersubjectively verifiable features; it goes outlaw, as it were, in that it generates its own rules. As Tustin exemplifies, a 12-year-old Elly developed a system based on the fact that certain numbers produced pleasant sensations and other numbers unpleasant ones (Tustin, 1986, 128–133). While numbers are thus “imbued” with tactile characteristics and categorized in this light, the common laws related to numbers become distant; the objective characteristics of numbers do not capture their rich idiosyncratic “feel.” Likewise, while preoccupied with the contrived idiosyncratic “shape” created when sitting on a chair, the potentially shared chair is not interesting *per se.* In this sense, like a self-sufficient material *hyle* devoid of intentional referent, autistic sensation shapes are “dead ends” (Tustin, 1986, 63; Tustin, 1990, 102), “sensation-dominated stop-gaps” (Tustin, 1990, 116), or, in Winnicott’s terms, a “cul-de-sac” (Winnicott, 1965, 183).

### 4.2. Autistic objects: the second skin

“Autistic objects,” too, are tactile formations considered apart from the actual objects that causally partake their formation. However, in contrast to autistic shapes, they are given rise by hard entities: “Autistic ‘objects’ are different from the soft, amorphous ‘shapes’ in that, as well as being hard, their outlines are rigid and static. They do not change as those of the malleable, fluid ‘shapes’ can do” (Tustin, 1986, 127). While “autistic shapes” function as tranquillizers, “autistic objects” primarily add to the invidual’s *sense of definition and boundedness*. Whereas the sensation shape alters when wriggling on the padded chair, the sensory constellation provided when squeezing a hard object only gets more intensive:

“At the beginning of treatment, an autistic 10-year-old boy called David used to bring a dinky car to every session. This car was clasped so tightly in the hollow of his hand that it left a deep *impression* when he took it out. […] Even if he placed it on the table, the deeply imprinted sensation remained […]. Another autistic child called Peter who was 6 years old at the beginning of treatment used to bring to his sessions a large keyring with over fifty keys on it. […] At the beginning of his treatment, it was the hard sensation in the hollow of his hand which was important to him” (Tustin, 1986, 102–104).

What solely matters is the impression. The situation can perhaps be illustrated in terms of an elastic *bubble* viewed from within. The inner surface of the bubble is all that matters: while the impression on the inner surface of the bubble is indeed caused by an external object pressed against its outer surface, the individual is not interested about this source, but is instead fully preoccupied with the immediate impression itself. Such “sensation-engendering activities” are neither accompanied with fantasies—in this sense, they differ from masturbation, Tustin notes (Tustin, 1992, 19; cf. 21). Instead of being directed, either in fantasy or in perception, outward, “attention is focused almost exclusively on endogenous bodily rhythms and sensations” (Tustin, 1992, 42). This not only temporarily averts attention from the external world, but altogether covers the latter. In the case of autistic objects, sensory awareness of an external object pressed against our skin is “replaced” by awareness of a set of sensory impressions on the skin (Tustin, 1986, 117; Tustin, 1990, 108–109). The latter assumes the position of the focal sensory object, and so what typically serves as an intentional pathway into the external world, is now a sensory dead end.

Autistic objects are thus experienced in a two-dimensional way: they are “surface impressions” without any “objective relevance” (Tustin, 1990, 42); “It [is] the impression that they [make] on body surfaces which [is] attended to. They [have] no significance in terms of actual, three-dimensional objects in the outside world” (Tustin, 1986, 302). These hard objects are “clutched or squeezed tightly so that they leave an impression behind” (Tustin, 1990, 40), and this impression is all that matters (Tustin, 1986, 302). And as autistic objects are defined and categorized “in terms of the hard sensations they engender” (Tustin, 1990, 17), it is understandable why they are not considered “in terms of their objective [and culturally defined] functions” (Tustin, 1990, 17): “meaning and function are not taken into account” (Tustin, 1986, 105). As Tustin exemplifies, David never playfully drove or made motor sounds with the toy car, and Peter never used his keys for opening doors or drawers. Instead of playthings, autistic objects “have a bizarre and ritualistic quality and the child has a rigidly intense preoccupation with them, which is not a feature of fantasy play” (Tustin, 1986, 103; see also Grandin, 1995, 35). Moreover, autistic objects are not considered in their uniqueness: if one of Peter’s keys were lost, “there was always another to replace it,” and “David did not bring the same dinky car each time” (Tustin, 1986, 104). Such “promiscuity” (Tustin, 1986, 104) adds to the assumption that it is the tactile impression that matters, not the object that in fact gives rise to it: “The tactile nature of an *object*—for example, whether it is hard or soft, rough or smooth, with sharp corners or rounded—is more important to an autistic child than its objective function” (Tustin, 1990, 52–53).

The collapse into the sensory “feel” has an important consequence. While squeezing the metal car entails sensations of a hard crust, shell, or armor (Tustin, 1986, 301; Tustin, 1990, 151), these sensations are not organized as sensory features *of an external object.* Tustin continues her clinical illustration:

“In working with [David] it became clear that the dinky car was felt to have magical properties to protect him from danger. As such, it was like a talisman or amulet. The difference between David’s car and a talisman was that he felt that by pressing it hard into the hollow of his hand it became a *hard extra bit to his body*” (Tustin, 1986, 102–103; *emphasis added*).

What Tustin means is that, in this special usage, the felt hardness is not experienced as the hardness *of the toy car*, but simply as *felt of hardness*—hardness that is felt *on one’s skin*. In clarifying this issue, Tustin speaks of “equation” (e.g., Tustin, 1972, 21; Tustin, 1986, 74; Tustin, 1990, 17–20; Tustin, 1992, 34–35). This concept comes from Hanna Segal, who distinguishes between “symbolic equation” and “symbolic representation” (Segal, 1981). In *symbolic representation*, the symbol is experientially *associated* with, but also *differentiated* from, what it symbolizes—e.g., an uttered or written word *stands for* X, but the word itself is not materially identical with X. In *symbolic equation*, by contrast, there is neither differentiation nor distance between the two; the symbol is not only taken to represent X but to *be* X. Just as in cases of “concrete thinking” one may feel that by hiding a note with the word “treasure,” one is actually hiding a treasure; and just as *thoughts of things* may be equated with things instead of being taken as subjective representations of things;^6^ so too, in the autistic individual’s “over-concretized mental functioning” (Tustin, 1986, 112; Tustin, 1990, 126), sensory impressions may be considered as *sensory objects themselves*, rather than *sensory appearances of* objects. In these lines, Tustin argues that autistic children “feel equated” with autistic shapes and objects “in a two-dimensional way” (Tustin, 1990, 17): “hard objects, such as toy engines and toy cars, that autistic children carry around with them, […] are experienced as parts of their body” (Tustin, 1990, 40).

Here we should expand on our earlier critical terminological note on “separateness.” Namely, to avoid a tempting misunderstanding, it is again crucially important to underline that *what* is experienced as “part of the body” (Tustin, 1986, 127; Tustin, 1990, 55) is not an object proper but the “surface feel” caused by an object proper. As Tustin puts it, one’s sensible surface “takes over the hardness of the object” (Tustin, 1990, 17). Importantly, what is “taken over” is neither the *hard object* nor the *hard surface of the object* (say, a thin layer of the latter), but the *felt hardness* (of the object) (see Tustin, 1992, 189). In all this, the external object as such (e.g., the toy car) is irrelevant. Like in the case of autistic sensation shapes, it is nothing but a “shape-producer” (Tustin, 1986, 122–123): the felt impression on one’s skin is all that matters. In short, the autistic child squeezes the hard object *to feel hard*. This felt hardness and definition serves to secure one’s boundedness and to preclude the unthinkable horror of “spilling out.” Rather than an “extra bit” on one’s body—which would re-introduce the setting with two adjacent depths—“autistic sensation objects” amount to what Bick calls “second skin” (Bick, 1968). Namely, given the localization of the impression on the skin, there is literally no distance between one’s felt boundaries and the autistic sensation object. Precaution is thus needed when talking of a relationship between the “two.” As Ogden puts it:

“The relationship to the object in this mode is certainly not a relationship between subjects, as in a depressive mode; nor is it a relationship between objects, as in a paranoid-schizoid mode. Rather, it is *a relationship of a shape to the feeling of enclosure*, *of beat to the feeling of rhythm*, *of hardness to the feeling of edgedness*” (Ogden, 1989, 32).

As I see it, Ogden’s point is that the beat and the sense of rhythm, for instance, can be *conceptually* distinguished from each other—i.e., the beat is what the sense of rhythm is “about”—but *experientially* the “two” are undifferentiated (see Taipale, 2021a, 208–213). Likewise, one can conceptually distinguish between the “feel” of the countering surface and the “feel” of the body surface on which the latter is localized, but in the tactile register of experience there is only “comforting oneness” (Tustin, 1990, 149), “the “ecstacy of ‘oneness”’ (Tustin, 1992, 121) or “continuity” (Tustin, 1986, 54). In this light, Tustin’s notes on toy cars, keyrings, and other hard objects being experienced as “parts” of one’s body (Tustin, 1990, 55, 79, 101, 108, 165, 218) or as “hard extra bits” to it (Tustin, 1990, 99, 165) indeed seem too careless. Indeed, in her theoretical framework, one can say that autistic objects “are not differentiated from the subject’s own body” (Tustin, 1990, 17), but it is nonetheless misleading to consider autistic objects as “hard extra bits,” for it is not *the hard bits* but *the hardness of the bits* that is experientially taken over. Differently put, the concept of “second skin” does not truly merit its name, for experientially speaking there is just *one* skin: once entrenched, these “me-extensions” (Winnicott, 1971, 135; Winnicott, 1986, 131) are no longer experienced as me-*extensions* but as undifferentiated me-*constituents*.^7^ The “autistic object is an object which is experienced as being totally ‘me”’ (Tustin, 1972, 62). This undifferentiation renders intelligible Tustin’s occasional notes where she associates separateness with “mutilation” (Tustin, 1990, 218): for what is experientially compromized or pulled off, is not only the “second” skin—the extra bits or the extra layer *on* the “first” skin—but one’s defining surface altogether.

## 5. The collapse of intentional depth

Sensory self-absorption—i.e., the use of autistic sensation shapes and autistic sensation objects—has two seeming advantages that together constitute the protective function of autism. Systematically summarizing these two “gains” serves to open the discussion on the experiential consequences this protective manouver.

First, the use of autistic objects and shapes *promotes* the sense of adhesion, *precludes* the sense of separateness, and thus *protects* the individual from the “unthinkable anxiety” of self-dissolution (e.g., Tustin, 1986, 143–144, 194, 208, 282; Tustin, 1990, 41, 107, 218):

“[Autistic] children feel that their skin surfaces ‘adhere’ to other surfaces in order to offset their terror of falling apart or spilling away. Autistic ‘objects’ meet the need. The autistic child presses a skin surface against the hard surface of an object, for example, a small car held tightly in the palm of his hand. The hard, well-defined cluster of sensations caused by this gives him a sense of bodily definition as well as making him feel secure and safe” (Tustin, 1986, 127).

As for the downsides, Tustin speaks of the “vicious circle” in which autistic shapes and objects “interfere with the perception of reality and are in turn intensified by not being modified by reality” (Tustin, 1972, 103; Tustin, 1992, 41–42). As she puts it elsewhere: “The more their attention becomes focused upon these autistic procedures, the more remote and strange the everyday world of ordinary people becomes” (Tustin, 1986, 132). While autistic sensation objects and sensation shapes “divert” attention away from the strange and frightening “not-me” (Tustin, 1986, 132; Tustin, 1992, 12) and thus have a protective function, they at once “insulate” the individual (cf. Winnicott, 1965, 187; Tustin, 1992, 189) from the intersubjective world. This is a matter of degree, however—after all, even if autistic shapes and autistic objects *per se* put the world “on mute,” as it were, “stimming” can make the world more bearable and thus *indirectly* facilitate sociality in autistic individuals.^8^

Second, autistic shapes and autistic objects promote a *sense of control* (e.g., Tustin, 1990, 37, 108–109). Auto-generated or “self-induced” sensation shapes and sensation objects are “felt to be instantly available” (Tustin, 1986, 121–137; Tustin, 1990, 108–109) and “in just the way the child wants them” (Tustin, 1986, 64, 112). As such, they appear almost identically every time, and hence are more reliable and predictable than the external environment can ever be. Yet, while the self-stimulating activity promotes “feeling of power” and the sense of “controlling things” (Grandin, 1986, 25), it also strengthens the vicious circle. For as soon as the use autistic objects and shapes “has become entrenched,” the actual caregiver “seems very unsatisfactory” (Tustin, 1986, 64; cf. Tustin, 1990, 66). Given that what is at stake is a “form of self-soothing that is ‘perfect’ in a way that no human can possibly be” (Ogden, 1989, 42), the caregiver can only appear as a failure—a situation that is very hard for the parent, we may add. Tustin follows Winnicott who describes autism in terms of “organization toward invulnerability” (Winnicott, 1989, 197–198; Winnicott, 1996, 220–221) and thus considers autism as a kind of premature declaration of independence. However, in fact, the child’s “beacon of orientation” (see Mahler et al., 1975, 7) is only relocated from the caregiving environment to the autistic sensations shapes and sensation objects (Tustin, 1990, 108), which is to say that the child becomes dependent on the latter instead (see Grandin, 1995, 34). As Tustin puts it, the individual not only “tyrannizes over” the autistic objects and shapes, but they also “tyrannize over him” (Tustin, 1986, 124): “The child felt that the existence of the magical ‘shapes’ depended upon his activities, and that *he depended upon their magical presence* to give him a sense of ‘being”’ (Tustin, 1986, 124; *emphasis added*).

Whereas sensation shapes and sensation objects, much like Winnicottian “subjective objects,” commonly function as kinds of *vitalizing* points of reference, that *nourish* our experiences of the shared environment, and thus convey the latter with a *personal* significance (Winnicott, 1965, 187; Ogden, 1991; Taipale, 2021b), in autism, the subjective “feel” no longer serves as such a pathway—instead, it gains a focal status *per se*, which complicates the individual’s experience of environment. Along with what could be called a *collapse of intentional depth*, the autistic individual is *caught on the surface.* As I will illustrate in the following, the word “depth” covers not only *concrete depth* but also what may be called *symbolic depth* and *intersubjective depth*.

For one, Tustin argues that, in autism, touch—i.e., the sensory modality promoting the sense of contiguity—“overrides” the other senses (Tustin, 1986, 120): “[Autistic children’s] main emphasis is on surfaces to which they can adhere in order to acquire some sense of bodily definition. Such a child lives mostly in a two-dimensional world” (Tustin, 1986, 56). Obviously, this is not to say that, in autism, the distal senses are simply inoperative (as if autistic persons would be blind or deaf) or that these other senses would each suffer something comparable to hemispatial neglect (as if autistic persons could only receptively *see* or *hear*, but not actively *watch* or *listen*). Instead, according to Tustin, the problem is fundamentally related to *sensory integration* and *intersensory coordination*. Tustin speaks of “insufficient” (Tustin, 1990, 107), “insecure” (Tustin, 1972, 105; cf. 86), or “loose” sensory integration in these children (Tustin, 1972, 49; Tustin, 1990, 141; cf. Tustin, 1992, 117), and argues that, in autism, “the normal integration of sense modalities seems to have been prevented” (Tustin, 1986, 63; for related contemporary contributions, see, e.g., McCleery et al., 2013, 39; Torres et al., 2013, 30; Whyatt and Craig, 2013, 225; Scheerer et al., 2021; Boldsen, 2022b). Whereas in normal cases each of the senses plays an irreplaceable role in overall sensory experience (e.g., sounds cannot be seen and colors cannot be touched), as *contiguity assumes a normative position* in the individual’s sensory experience, the boundaries between the different senses remain unclear (see Tustin, 1992, 126). That is to say: the “integration of touch and sight has been halted” (Tustin, 1986, 63), and “seeing and hearing are often experienced as by the child in a tactile way as being *touched* by the object” (Tustin, 1992, 126). Due to the “over-valuing tactile physical contacts and the sensations thereby aroused” (Tustin, 1990, 102), “everything seems to be ‘experienced in a tactile way’ (Tustin, 1990, 218, 52–53).” Tustin exemplifies:

“vision and hearing, as a result of the undue dominance of the sense of touch, become excessively imbued with tactile sensations. […]. [I]f they [autistic persons] see something unpleasant, it feels as if their eyes are being struck by a painful object, while a loud noise can be felt as a blow on the ear” (Tustin, 1986, 145; Tustin, 1992, 126).

There are various ways to interpret this “sensory override,” however. Looking at the extremes serves to underline the point I want to make.

For one, if the claim about “two-dimensionality” is taken *literally*, Tustin seems to be suggesting that some autistic individuals *believe they are actually touching what they see.* Some of Tustin’s claims clearly favor the literal interpretation. For one, she notes that some of her autistic patients “feel that their eyes,” much like their hands, “are physical instruments to control objects” (Tustin, 1986, 145). She also gives a clinical vignette of a bewildered autistic child who, in perceiving a letterbox (near to her) and a man (farther away in the same direction), wonders out loud *why the letterbox is bigger than the man* (Tustin, 1990, 52). Along with “the breakdown of the ‘long-distance’ sense[s]” (Maiello, 2015, 41), it is as if *all* sensory data—and not just tactile data—was equally considered as being at *zero-distance*. And as everything is thus taken in its “face value,” what remains is a “flat, two-dimensional world without perspective” (Tustin, 1990, 52): hence the seen letter box not only *seems* bigger, but is *believed* to be bigger. However, while theoretically conceivable, the obvious problem with the literal interpretation is that the same autistic child nonetheless successfully walks into Tustin’s counselling room, can approach and grasp objects that she is not yet in touch with, and hence readily displays awareness of three-dimensional space.

On the other interpretative end, “two-dimensionality” is viewed as a *metaphor*, and the claim of touch “overriding” the other senses is understood in terms of *heightened tactile affordances.* To be sure, coming out of the sauna in the winter time and approaching the hole in the ice, the *seen* water may readily give me the chills before I *tactually sense* the water. However, there is an obvious experiential difference between anticipating a feeling of the water and actually feeling the water—for sure, here the coldness of the seen water is not yet tactually felt. Like in cases where certain visual qualities are *associated* to certain tactile impressions (e.g., red is a “warm” color, and light blue a “cold” one), here too there is a metaphoric distance between the visual and the tactile—the former “refers” or “alludes” to the latter, but is not readily “imbued” or “saturated” by it. Relatedly, it is worth noting that, in Tustin’s example quoted above, the feeling of “being struck” is localized on *the eye*, and not on some other area of one’s body or on the body overall—and it is not uncommon to hear autistic individuals saying that looking at something unpleasant or disturbing *hurts their eyes*. In the water example, by contrast, the seen water typically conveys an expectation of coldness *all over the body*, perhaps giving emphasis to the most sensitive body parts—and such experiences are quite different from ones where the coldness of the water is localized on the eye.

Accordingly, whereas the first interpretation (suggesting that individuals feel they are *actually touching* the seen object with their eyes or with their gaze) seems *too strong*, the second interpretation (where the seen object is merely considered to be awakening *lively expectations* of particular tactile impressions) seems *too weak.* Indeed, as often is the case, the truth lies between the extremes. However, in this case, there may be several truths: namely, *in some cases* of autism, two-dimensionality may be taken quite literally and comprehensively, whereas *in other cases*, a metaphoric and relative interpretation might be more suitable. As I see it, this flexible interpretation suits well with the contemporary idea of autism as a *spectrum*, while also doing justice to Tustin’s rather heterogenous sample of patients. Moreover, as noted already at the outset, while Tustin herself mainly focuses on the severe end of the spectrum, much of what she says may also be applicable, *mutatis mutandis*, to the high-functioning end of the spectrum as well.

This flexible interpretation may be complemented by considering the “tactile override” as an *ontological* issue. Namely, instead of referring to a *belief that distal objects are in immediate proximity*, or to an *expectation of specific tactile impressions arising on the basis of visual data*, touch can also be viewed as “overriding” the other sense modalities in the sense of “monopolizing” its position as the *measure for reality*: “only what is tangible and physically present is felt to exist” (Tustin, 1986, 63; Tustin, 1990, 102). To be sure, as already said, touch plays a fundamental ontological role also in neurotypical sensory experiences—for seen things to appear as real, they must appear as *tangible*, and if not, they are destined to appear as hallucinations. Here the requirement of *potential touch* seems to be replaced by the requirement of *actual touch*. Tustin’s idea seems to be that, while distal objects do not meet this requirement, despite being perceptually registered, they seem to be less real, as it were: much like recollective images or passing thoughts, they do not motivate spontaneous bodily responses, but swerve in the unintegrated background of awareness. If this is the case, picking out a particular set of impressions into the spotlight of awareness may be extremely laborous. As Naoki Higashida, a 14-year-old autistic child, communicates:

“A person who’s looking at a mountain far away doesn’t notice the prettiness of a dandelion in front of them. A person who’s looking at a dandelion in front of them doesn’t see the beauty of a mountain far away. To us, people’s voices are a bit like that. It’s very difficult for us to know someone’s there and that they’re talking to us, just by his or her voice” (Higashida, 2013, 47–48).

Related descriptions seem to be fairly common—for example, as one autistic adult person tells to an interviewer: “If we were in a restaurant and there was a discussion going on in the next table, the conversation in that table would enter my awareness just as intensively as your voice” (Rossi, 2023, A13; *my translation*). What seems to be blurred, along with the sense of depth, is the foreground/background distinction.

Moreover, while touch gains a dominant ontological position, a certain *disproportion* is introduced into the structure of affordances or sense of environmental possibilities. Namely, whereas *references from the visual to the tactual* may be heightened, *references in the opposite order* tend to be weakened—even to the extent of becoming redundant. As said, in typical cases, a sudden knock on one’s shoulder tends to motivate turning around in search for additional visual data. Yet, along with the monopolization of touch, vision might not seem to add anything relevant to the experience—and hence turning around would be a redundant act instead of something spontaneously motivated. In such circumstances, reacting in a neurotypical manner might require a laborous deliberate effort—and autistic persons are known to engage with such “masking” behaviors to fit in. As I see it, this relates to the heightening or lowering of the sense of touch in autism. As touch becomes the measure for reality, reality is prone to emerge suddenly, abruptly, and unexpectedly—as if out of nowhere. It is perhaps helpful to compare the experiential situation with that of blind persons, who cannot see material objects approaching (before being already in touch with them), or with that of traumatized individuals, who are equally unable to foresee when or where a horror-triggering experience presents itself (until it already does). In both cases, objects seem to emerge out of nowhere and recede back into this “engulfing ‘nothingness”’ (see Tustin, 1990, 218; Karlsson, 1996).^9^ Regardless of the obvious differences between these cases, and regardless of questions of severity, the comparison is helpful, insofar as autistic individuals, too, tend to remain particularly alert and on guard vis-à-vis the potentially impinging surroundings. Indeed, if the capacity of the distal senses to awaken tactile anticipations and practical affordances is more or less compromized, the emerging tactile sensations (e.g., being touched) are prone to be surprising and potentially startling—in their unpredictability, they constitute a threat that prompts constant vigilance and alertness. It may be that the heightening and lowering of the sense of touch are two sides of the same issue, as tactile numbness could be considered as a reaction to an overtly intensive tactile stimulation.^10^

Here we come back to where we started from. Namely, what is disrupted by two-dimensionality—now taken either in *more literal* or in *more metaphoric* sense—is the sense of “insides.” In this regard, the drawings of autistic children are revealing: “David used to draw two-dimensional cars with the front, back, and sides all shown, and he used to puzzle over them. He could not grasp three-dimensionality” (cf. Meltzer, 1975, 299; Tustin, 1990, 138); another child “would draw the back of a house on the reverse side of the paper on which he had drawn the front elevation of the house,” while yet another child would go up to a picture of hills hanging on the consulting-room wall and turn the picture over to see what was on the backside of the hills (Tustin, 1986, 301). Tustin concludes that, while living “in terms of surfaces,” autistic children “are not aware of the inside of objects” (Tustin, 1990, 41). This also carries over to experiences of being inside something. Just consider the experience of a child who is hiding *in here* (say, under a blanket), knowing that seeker is *out there* (beyond the blanket). Now if, along with the tactile modification, the felt inner surface of the blanket defines the parameters of one’s experiential reality, the idea of being “contained” by the blanket would be as alien as the neurotypical person’s feeling of being “contained” by one’s skin: for what matters in both cases is the immediate “feel” of a “limiting membrane” (see Winnicott, 1958, 230), and not the sense of opposition with what this interface insulates oneself *from*. Tustin exemplifies:

“If he goes inside a box, or a tunnel, or a cupboard, it is the sensation of being hidden and protected which is significant to him. It is not the going inside. For most of the time, he has little awareness of the difference between outsides and insides” (Tustin, 1986, 56).

In a certain sense, such experiences are familiar from normal everyday life as well. Just consider the experience of pulling the blanket over your body when going to bed, whereby it might be emphatically the sense of self-definition that is important, not the blanket as such; or consider the way in which you may emphatically use music as a *wall of sound* or a “sound bath” (see Anzieu, 1979, 30–32), whereby the melodic and rhythmic structure of the song may be less relevant. When covered by such a *sonorous blanket*, if you will, what matters is the sense of definition or “self-envelopment by lulling shapes” (see Tustin, 1986, 123) and not the sense of being *inside* the walls of sound. The difference with autistic experience is that the other modes of experience, where the blanket or the music are attended in their own right, are not only temporarily outshined but more comprehensively “impeded” or “blocked” (Tustin, 1986, 37, 110; see also Ogden, 1989, 46).

The idea of preoccupation with the immediate “feel” or the phenomenal “face value” extends beyond questions of concrete depth and spatial interiors. For one, if Tustin is right when assuming that, in autistic experience, “the ‘shapes’ of sound, smell, taste and sight [seem] to be ‘felt’ rather than heard, smelled, tasted or seen” (Tustin, 1986, 120), cases where autistic children do not turn to look when being spoken to may be understood in the light of the claim that it is not first and foremost the *content of the words* but the *sensory “shape” of the call* that enthralls or captivates them—like often with music, *what* is expressed is less important than *how* the expression proceeds (see Taipale, 2023a). In effect, what is flattened besides the *spatial depth* is also the *symbolic or expressive depth*. To illustrate, when being addressed, our focus is usually not on the *expression*, but on the *meanings* to which the heard words and sentences serve as expressive pathways—as Merleau-Ponty puts it: “expression [normally] fades out before what is expressed, and this is why its mediating rôle may pass unnoticed” (Merleau-Ponty, 2002, 466). *Vice versa*, if the materiality of the expression gains prominence, our access to the meaning is hindered (see Taipale, 2015).

Again, this is a matter of degree. In an extreme case, the sensory “feel” is all that matters, and the meaning of words remains out of sight. Tustin notes that of autistic children who speak, fairly many are “echolalic” (Tustin, 1972, 111; Tustin, 1986, 57; Tustin, 1990, 35, 134): they repeat words, as if *trying out* how the phonemic shapes feel in their mouths and ears.^11^ As a sensory dead end, the sensory shape or phonemic contour of the words thus outshines the contained symbolic meaning (see Tustin, 1990, 135). On the other end of the spectrum, attention may also be stuck in the “face value”—yet not in the sense of *the material composition of the expression*, but in the sense of *the concrete literal meaning*. Preoccupation with the *face value meaning* would partly explain why social cues, implicit norms, unwritten rules, metaphors, hidden meanings, pretense and lying prove complex to autistic persons. As Clara Törnvall, a high-functioning autistic author, puts it:

“a successful metaphor carries oneself from one world to another so that a kind of double exposure is introduced, where both worlds are simultaneously visible. Instead of double exposure, all I experience is a paradox; the metaphor only makes things more opaque. This is because of my tendency to focus on details” (Törnvall, 2023, 92, *my translation*).

Törnvall further explains that hidden social cues, irony, sarcasm, and metaphors are difficult to her, because she takes symbols literally and is “not able to cross the gap between abstract and concrete without having to ponder about it” (Törnvall, 2023, 92). The underlying conventions are not spontaneously accessible to her. Instead of being automatically directed at the same conventional meaning as the neurotypicals, in autistic person’s case the associative dimension is a “lonely place” (Törnvall, 2023, 93). As Törnvall exemplifies, it has been commonly agreed that a “table’s leg” is just a figurative term for specific part of the table, and that calling someone a “pig” is simply meant to convey the opinion that this person is unpleasant or misbehaving, but Törnvall herself cannot help but first taking these descriptions literally, in their “face value”—as absurd claims about a table having *legs* and about certain persons being representatives of the *Sus domesticus* genus! Just as there is a *gap* between immediate sensory “feel” and the shared multisensory object, there is a *gap* between the immediately grasped literal meaning and the jointly agreed metaphorical meaning—and in both cases, bridging the gap proves extremely laborious.

The preoccupation with the immediate “feel,” with the expense of what the “feel” is about, applies equally to interoceptive experiences. Tustin argues, for instance, that autistic children are often “unaware of sensations with more normal objective relevance,” and hence “many of these children are unaware of being hurt if they fall down” (Tustin, 1990, 218). Törnvall’s autobiographical descriptions foster this hypothesis: “I am not connected with my body. My periods surprise me every time, and if I became fatally ill, I would not make it to the hospital on time, because I would not notice that I am ill. […] I do not notice hunger either […]. It is not that I am trying to lose weight; I simply forget to eat” (Törnvall, 2023, 58).

Besides *symbolic depth*, being caught on the surface thus relates to problems in accessing *intersubjective depth*, or the expressive depth of another person. If everything is condensed to the awakened sensory “feel,” it is understandable why autistic children commonly face problems in grasping or “reading” the minds of others—minds that “contain” thoughts and ideas that are not immediately graspable by the senses. Like with “word bodies,” so too the expressive body of the other person at first catches one’s attention either in terms of the immediate “feel” or in terms of the “face value meaning.” While the former complicates awareness of other sensing beings as such, the latter makes it difficult to track exaggeration, lying, and any indirect social cues. Likewise, the autistic person’s own expression tends to be straightforward and honest: “autistic persons use language for the purpose of transmitting knowledge, not in order to impress someone” (Törnvall, 2023, 88). For Törnvall, embellishing the truth (e.g., exaggerating or lying) feels as if she is hurting herself (Törnvall, 2023, 89), and this is presumably because she is intensively aware of the disproportion between her spontaneous thought in its face value and what she gives out to others.

In this light, we may hypothesize that “deficits in the ability to initiate and to sustain reciprocal social interaction and social communication” (ICD-11) might be fundamentally owing neither to a lack of empathy nor to a heightened awareness of other minds, but to an unusual modification of tactile modality, whereby the individual is *caught on the surface*. In the light of what has been put forward here, modifications of empathy and the theory of mind may be owing to modifications in the tactile modality. Likewise, the “restricted, repetitive, and inflexible patterns of behavior, interests or activities” (ICD-11) may not be a sign of a collapse of affect-regulative capacities, but a reaction to problems given rise by the aforementioned modifications and the heightened need for adhesive sense of being. As said, according to Tustin, the latter is something that the autistic individual pursues to maintain by making use of autistic shapes and autistic objects.

## 6. Conclusion

I have here clarified Tustin’s theory of autistic experience and argued that, according to her, autism centrally involves a disturbance in sensory integration, which leads to what I called a *collapse of intentional depth*. I first examined how touch, in collaboration with the other senses, typically opens into a dimension of depth. Analyzing how such opening may be directed outward or inward, I distinguished between “exteroceptive depth” and “interoceptive depth.” I then argued that, according to Tustin, the Kleinian setting with the “two depths” is developmentally preceded by an “adhesive” sense of being—a mode of experience maintained by a sense of immediate (tactile) contact. Analyzing how threats to the adhesive sense of being entail horrifying experiences of leaking off, I argued that Tustin considers autistic experience as a protective reaction against such unthinkable anxieties. As I illustrated, this protective maneuver entails a collapse into a “two-dimensional” mode of experience, where sensory attention snuggles into the immediate “feel”—the subjective “face value” of the object. Illustrating the explanatory potential in Tustin’s account, I argued further that the collapse of “depth” covers not only *spatial depth*, but *symbolic depth* and *intersubjective depth* as well. By examining the different facets of the collapse of intentional depth, I tried to show how Tustin’s conceptualizations convincingly render intelligible certain commonly recognized features of autism, such as deficits social interaction, repetitive patterns of behavior, and atypical use of objects.

The possible theoretical and clinical relevance of my interpretation remains to be seen. As said, if Tustin’s account is viewed as an *reductionist etiological theory*, according to which autism is *exclusively caused* by psychodynamic factors (rather than neurological and/or genetic factors), her account appears hopelessly outdated. However, as I have suggested, this reading would grossly miss Tustin’s point. What she offers instead is a *non-reductionist psychodynamic approach* to autistic experience—a theory whose validity carries no reductionist commitments. As I have tried to illustrate, this non-reductionistic approach draws Tustin out of the margins and brings her conceptualizations into communication with contemporary multidisciplinary autism research—in particular, it communicates with those contemporary accounts that underline the role of tactile modification at the core of autism (see, e.g., Cascio et al., 2012; Mammen et al., 2015; Boldsen, 2022a), while also putting pressure on the theory of mind paradigm in autism research. Assuming that the etiological grounds are not mutually exclusive but complementary, what is required is not a *choice* but *collaboration*—and this is what Tustin, too, was insisting upon. After all, regardless of whether the exceptionally strong drive to integration (Tustin, 1992, 19), heightened sensitivity (Tustin, 1990, 218), or the over-valuing of the tactile domain (Tustin, 1990, 102) are causally owing to neurological, genetic, psychological issues, or to a combination of them, understanding autistic experience is an important task *per se.* Besides purely theoretical purposes, such understanding is needed to improve clinical practices and also to pinpoint poorly functioning societal structures that neglect this unique mode of experience, thus unnecessarily burdening the life of autistic individuals. After all, to end with Törnvall: “The most difficult thing for mildly autistic persons is that they are, so to speak, too normal for their challenges to be taken seriously, but on the other hand, too deviant to fit in” (Törnvall, 2023, 116).

## Data availability statement

The original contributions presented in the study are included in the article/supplementary material, further inquiries can be directed to the corresponding author.

## Author contributions

The author confirms being the sole contributor of this work and has approved it for publication.

## Footnotes

1 As a side note, *which one* of the depths gains experiential prominence might be partly explained not only by one’s interest but also by the affective nature of the tactile sensation itself—I have elsewhere suggested that pleasant sensations tend to open tactile experience outward, whereas unpleasant sensations tend to turn us inward (see Taipale, 2023b). It would also be interesting to link this issue with certain empirical studies, but that must be left for further studies (see Cascio et al., 2012).

2 With “mutually differentiating opposition,” Ogden refers to the Kleinian “depressive position” and, and with “merger,” to the “schizo-paranoid position.”

3 I am obliged to Elina Havukainen at *Autism Finland* for making me aware of this.

4 As for pleasant examples of sensory self-absorption, one could refer to an orgasm, for instance.

5 It might be interesting to compare Tustin’s concept of “shape” with Merleau-Ponty’s concepts of “schemas” and “Gestalts.” Merleau-Ponty’s notes, for instance, that the “*Gestalt* of a circle is not its mathematical law but its physiognomy” (Merleau-Ponty, 2002, 70). Such comparisons, however, will be left for other studies.

6 Tustin interestingly argues that insofar as thoughts feel thing-like, the experience of *forgetting* may come close to the experience of parts of oneself leaking out (Tustin, 1986, 199–201). It might be interesting to develop such ideas toward the *extended mind* hypothesis, for instance, but this will be left for further studies.

7 A comparison with what phenomenologists call “incorporation” is perhaps helpful: certain external objects like equipment may be organized as part of one’s body—for instance, rather than experiencing one’s pleasant clothes as being wrapped *around one’s body*, it is rather that the environment is experienced as being around *one’s clothed body*. However, what is “incorporated” in our case are not (thin) three-dimensional clothes, but the two-dimensional sensory feel that arises when in tactile contact with the latter.

8 I am grateful to Elina Havukainen for a related note on this issue.

9 I am obliged to Henrik Enckell for this comparison.

10 This might suggest that an heightened sensitivity is more common in the high-functioning end of the spectrum, whereas lowered sensitivity is more common in the more severe cases. Such developments, however, will be a matter of further studies. I want to thank Eeva Pihlaja for discussions related to this matter.

11 Tustin’s ideas might also be compatible with the autobiographical descriptions of Higashida, who says he repeats the questions and utterances of others in order to *hold on* to the thought in question (Higashida, 2013, 29–30). To be sure, here too, we must be careful not to make too strong claims. After all, like with “stimming,” especially at the high-functioning end of the autistic spectrum echolalic behaviors most likely have merely a *relative* status: while modifying the individual’s attention vis-à-vis the environmental stimuli, they may at the same time function as facilitators of interaction (both by making the situation more tolerable and possibly also by enhancing one’s capacities in “holding on” to fleeting thoughts).
